# Three-Dimensional Melted Electrowriting Drug Coating Fibers for the Prevention of Device-Associated Infections: A Pilot Study

**DOI:** 10.3390/bioengineering11070636

**Published:** 2024-06-21

**Authors:** Joana P. Martins, Elisabete T. da Silva, António A. Fernandes, Sofia Costa de Oliveira

**Affiliations:** 1 Associated Laboratory for Energy, Transports and Aerospace (LAETA), Institute of Science and Innovation in Mechanical and Industrial Engineering (INEGI), 4200-465 Porto, Portugal; jamartins@inegi.up.pt (J.P.M.); mesilva@inegi.up.pt (E.T.d.S.); aaf@fe.up.pt (A.A.F.); 2 Faculty of Engineering, University of Porto, 4200-465 Porto, Portugal; 3 Department of Pathology, Faculty of Medicine, University of Porto, 4200-319 Porto, Portugal; 4 Center for Health Technology and Services Research—CINTESIS@RISE, Faculty of Medicine, University of Porto, 4200-319 Porto, Portugal

**Keywords:** melt electrospinning, device-related infections, biofilm formation, drug release, biodegradable implants, PCL

## Abstract

Medical device-related infections (DRIs), especially prevalent among critically ill patients, impose significant health and economic burdens and are mainly caused by bacteria. Severe infections often necessitate device removal when antibiotic therapy is inefficient, delaying recovery. To tackle this issue, PCL drug-eluting coated meshes were explored, and they were printed via melt electrowriting (MEW). These meshes were coated with gentamicin sulfate (GS) and tetracycline hydrochloride (TCH) and underwent FTIR analysis to confirm drug integration. Antimicrobial activity was assessed via agar diffusion assays and biofilm formation assays against bacterial strains: *Pseudomonas aeruginosa* ATCC 27853, *Escherichia coli* ATCC 25922, *Staphylococcus aureus* ATCC 43300, and *Staphylococcus epidermidis* ATCC 35984. FTIR analysis evidenced the presence of the drugs in the meshes. TCH displayed broad-spectrum antimicrobial activity against all strains, whereas GS was effective against all except *S. aureus*. These findings indicate the potential of cost-effective ultra-fine drug coating fibers for medical device applications, offering infection prevention during implantation. This preliminary study demonstrates the feasibility of producing drug-eluting fibers for DRI prevention through a non-toxic, fast, and cost-efficient technique, paving the way for enhanced patient care and reduced healthcare costs.

## 1. Introduction

The implantation of medical devices in a patient’s body, as is the case of pacemakers and catheters, can lead to the development of device-related infections (DRIs). Previous studies have shown that DRIs account for 45% of all hospital-acquired infections, with 12% of patients with indwelling urinary catheters developing catheter-associated urinary tract infections [1,2]. DRIs affect not only patients with a weaker immune system due to previous medical conditions but also healthier patients. In many instances, the contamination of a medical device, leading to the development of DRIs, most likely occurs via inoculation with microorganisms from the patient’s skin during insertion due to contamination by medical staff or due to incorrect device sterilization. These infections lead to high morbidity and mortality, depending on the state of the patient and the implanted device, requiring new treatments and, depending on their severity, the removal of the device, with added costs for patients and healthcare systems [3,4].

DRIs are most commonly caused by bacteria and fungi capable of adhering to the surface of the devices and developing biofilms. The limited penetration of antimicrobials into the biofilm and the subsequent entrapment or inactivation of these antimicrobials lead to antibiotic tolerance and resistance, which poses a great obstacle to the treatment of such infections [5]. If the infection cannot be treated with antibiotics, it can lead to the permanent removal of the medical device, which may not be feasible or desirable. This way, preventing biofilm formation becomes a priority for preventing DRIs [6,7]. Even though preventive strategies have already been established, these are not enough to avoid DRIs, leading to continued research in this field for new ways of prevention and treatment. In order to decrease the incidence of DRIs, various guidelines have been published, such as national evidence-based guidelines for preventing healthcare-associated infections in NHS hospitals in England, where different recommendations are given regarding the sanitation of the environment and the devices, as well as the use of personal protective equipment [8]. However, these preventive guidelines are not enough to stop infections from developing, leading to the need for additional treatment with antibiotic therapy. Antibiotics can prevent the formation of biofilm since planktonic bacteria are killed and/or prevented from adhering to the surface [6,9]. The possibility of incorporating antimicrobials with medical devices has been explored over the years with the intent of preventing the adhesion of microorganisms to prevent the formation of biofilms. Minocycline–rifampicin (MR)-coated catheters have shown highly potent antibacterial activity against key Gram-positive pathogens and some Gram-negative pathogens [10].

The application of additive manufacturing (AM) techniques to produce novel medical devices as a strategy to prevent and treat DRIs has been researched before, as in the case of drug-eluting catheters printed via a fused deposition modeling (FDM) process [11,12]. AM comprises different types of techniques, one of them being electrospinning (ES). ES is based on the principle of an electric field that is created by a potential difference between the spinneret and the collector, pulling the polymer solution or melt from the spinneret, allowing for the manufacture of ultra-fine fibers [13,14]. In this process, the high voltage applied to the polymer solution creates instabilities in the jet, making it more difficult to control the deposition of the fibers, contrary to many traditional AM techniques. However, melt electrospinning writing or melt electrowriting (MEW) uses a polymer melt instead of a polymer solution, which does not suffer from these instabilities, allowing for controlled deposition. Additionally, the use of a polymer melt avoids the use of toxic solvents to produce the fibers, contrary to ES [15,16,17].

Different studies have shown that tetracycline hydrochloride and gentamicin sulfate are two antibiotics that can be applied in DRI treatments through incorporation with polymers. The former exhibited antimicrobial activity against *Staphylococcus aureus* when integrated into a thermoplastic polyurethane (TPU) matrix [11], and the latter was incorporated with polylactic acid (PLA) to inhibit *Staphylococcus aureus* and *Staphylococcus epidermidis* activity [18].

Thus, the research reported was focused on the incorporation of the mentioned antimicrobials with the reduced-diameter fibers produced via MEW, which has advantages in producing medical devices, such as implant meshes. However, the results of drug release mechanisms studies can be generalized to other medical devices, such as catheters, which have dimensions on the order of micrometers [19].

The aim of this research was to produce PCL drug-coated fibers and understand their antimicrobial effects through a fast and low-cost technique that has not been widely explored for treatment strategies against DRIs.

## 2. Materials and Methods

### 2.1. MEW Printing of Medical-Grade PCL Meshes

**Figure 1:**
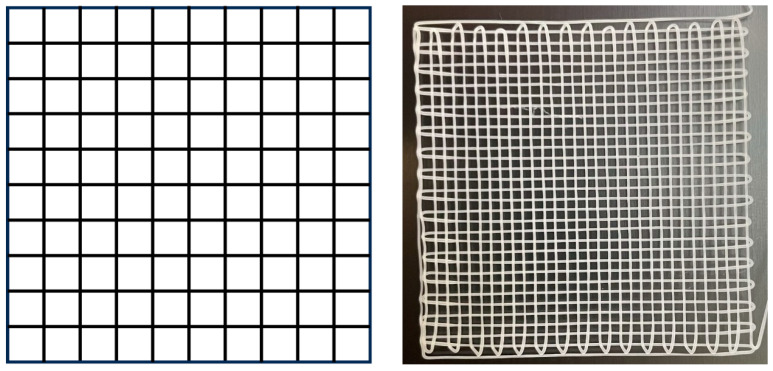
Square mesh design and geometry.

Medical-grade PCL with an inherent viscosity of 1.00–1.30 dL/g was obtained as a commercial product from CORBION (NL) known by the trade name PURASORB PC12. Once the printing parameters were optimized, as shown in Table 1, meshes with 69 × 69 mm and 1.5 mm of horizontal and vertical space between fibers (Figure 1 presents mesh geometry) were printed using a Melt Electrowriting Prototype developed with a “SPINMESH” research project, completed in 2021.

The preliminary fiber diameter was set to 319 μm. The extrusion rate of the PCL polymer in the g-code was estimated using empirical Equation (1), where E represents the polymer to be extruded, D is the intended diameter for the fiber, and L is the length of the print segment.

(1) $$E=\frac{{\left(D\times 1.5\times 1.13\times 0.001\right)}^{2}}{1.75^{2}}$$

### 2.2. Preparation of Meshes Loaded with TCH and GS

Tetracycline hydrochloride and gentamicin sulfate were purchased from Sigma-Aldrich (St. Louis, MO, USA). To incorporate the antimicrobials, 69 × 69 mm meshes were cut into 1 cm^2^ meshes. Then, they were sterilized for 30 min under UV-C light in a Thermo Scientific MSC-Advantage Class II biological safety cabinet (Thermo Fisher Scientific, Waltham, MA, USA). Solutions with 1% *w*/*w* concentration of GS and TCH were prepared by dissolving the antibiotics separately in 50 mL of distilled water. After sterilization, the meshes were dipped in the solutions for 24 h at room temperature and afterward air-dried for 24 more hours in the cabinet.

### 2.3. FTIR Analysis of the PCL-TCH and PCL-GS Meshes

Fourier Transform Infrared (FTIR) analysis was conducted using an Agilent Cary 360 FTIR spectrometer from Agilent Technologies (Agilent Technlogy, Santa Clara, CA, USA) to evaluate the presence of TCH and GS in the meshes after incorporation. The infrared spectra were documented in a spectral range from 4000 cm^−1^ to 650 cm^−1^, with an 8 cm^−1^ resolution and a total of 32 sample scans. FTIR analysis was performed on three samples for each antibiotic as well as for a control sample. The spectra were then assembled using the average values of the three readings [20].

### 2.4. In Vitro Microbiological Assays

*S. aureus* ATCC 43300, *Staphylococcus epidermidis* ATCC 35984, *Escherichia coli* ATCC 25922, and *Pseudomonas aeruginosa* ATCC 2785 were purchased from the American Type Culture Collection (ATCC, USA). *S. aureus*, *E. coli*, and *P. aeruginosa* were cultured in Brain Heart infusion broth (BH), and *S. epidermidis* was cultured in Luria–Bertani broth (LB) overnight in an orbital shaker at 37 °C and 120 rpm.

#### 2.4.1. Zone Inhibition Assay

A suspension of 0.5 MacFarland bacteria was smeared in triplicate on a Mueller–Hinton agar plate. The PCL-TCH and PCL-GS meshes and unloaded meshes with 1 cm^2^ were pierced and sterilized for an hour (30 min on each side) under UV-C light. Then, they were placed on the plates containing the strains *S. aureus* ATCC 43300, *S. epidermidis* ATCC 35984, *E. coli* ATCC 25922, and *P. aeruginosa* ATCC 2785 and incubated for 24 h at 37 °C. The antibacterial activity was determined by measuring a clear inhibition zone in mm.

#### 2.4.2. Bacterial Biofilm Assays

The PCL-TCH and PCL-GS meshes were plated in a 12-well polystyrene plate well. The antimicrobial efficiency was measured using a concentration of 1 × 10^7^ cfu/mL of bacterial suspension. With one ml of each strain suspension per well in the 12-well plate, and after 24 h of static culture at 37 °C, the material sample at the bottom of the well was collected. After gently rinsing the sample three times with phosphate-buffered saline (PBS) buffer, the material samples were placed in a sterile glass test tube (1 per tube), and 1 mL of BH medium was added. To separate the microorganisms adhering to the surface of the material, the tubes were sonicated in an ultrasonic bath for 2 min. The suspension was serially diluted, and 100 µL of the suspension was plated in BH and Sabouraud agar for 24 h at 37 °C for colony-forming units (CFUs) enumeration. All of the experiments were run in triplicate.

### 2.5. Scanning Electron Microscopy (SEM)

To evaluate the cell morphology of the microorganisms after biofilm formation in PCL drug-coated meshes of PCL-TCH and PCL-GS, a JEOL JSM 6301F/Oxford INCA Energy (Oxford Instruments, Abingdon, Oxforshire, UK) 350 high-resolution Scan Electron Microscope with X-ray microanalysis and a FEI Quanta (SEMTech Solutions Inc., North Billerica, MA, USA) 400 FEG ESEM/EDAX Genesis X4M high-resolution (Schottky) Environmental Scanning Electron Microscope with X-ray Microanalysis and Electron Backscattered Diffraction analysis were used with a 15 mA current. The mesh specimens were fixed and coated with Au/Pd for 120 s prior to SEM imaging.

### 2.6. Statistical Analysis

The collected data for biofilm formation underwent analysis using Microsoft Excel version 2308 using a *t*-test. Statistical significance was attributed to *p*-values equal to or less than 0.05.

## 3. Results

### 3.1. Preparation of Meshes Loaded with TCH and GS

**Figure 2:**
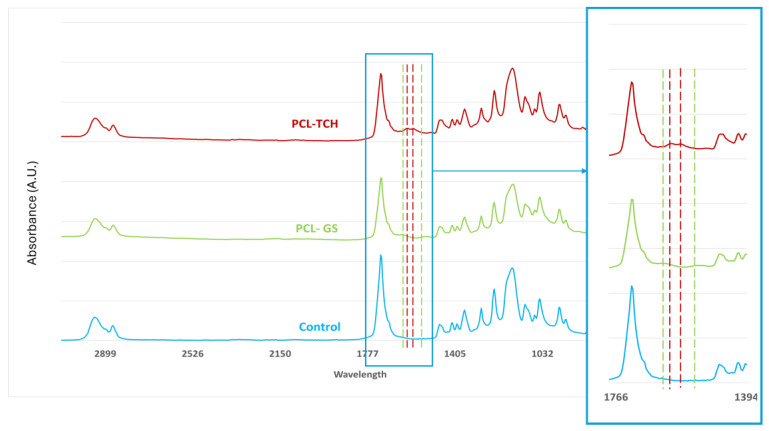
FTIR spectra of PCL-TCH and PCL-GS medical-grade PCL meshes. Color dotted lines show the presence of the representative peaks of each antimicrobial drug, comparatively to PCL spectra (control).

The infrared spectra obtained from FTIR analysis (Figure 2) confirm the incorporation of both TCH and GS in their respective meshes. In both PCL-TCH and PCL-GS spectra, it is possible to detect characteristic peaks of PCL, also evidenced in the PCL spectra: 2940 cm^−1^, 2866 cm^−1^, and 1722 cm^−1^, corresponding to -CH2 asymmetric stretching, -CH−CH2 symmetric stretching, and C=O carbonyl stretching [21,22,23]. Regarding the PCL-TCH meshes, two characteristic bands are visible: 1610 cm^−1^ for the C=O stretching in rigs A and 1576 cm^−1^ for the NH2 amide [22,23], proving the incorporation of TCH. Concerning the PCL-GS meshes, it is possible to identify two characteristic peaks, 1632 cm^−1^ and 1532 cm^−1^, related to the N-H bending vibrations of aromatic amine present in gentamicin [24,25], confirming its presence.

### 3.2. In Vitro Microbiological Assays

#### 3.2.1. Zone Inhibition Assay

**Figure 3:**
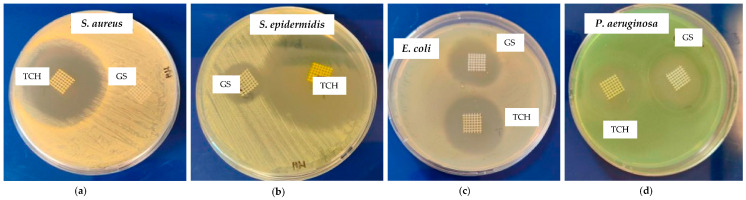
Inhibition zone of PCL-TCH and PCL-GS meshes against *S. aureus* (**a**), *S. epidermidis* (**b**), *E. coli* (**c**), and *P. aeruginosa* (**d**).

Figure 3 presents inhibition zones for the PCL-TCH and PCL-GS meshes against *S. aureus*, *S. epidermidis*, *E. coli*, and *P. aeruginosa*. Table 2 summarizes the values of these diameters and for control meshes (only PCL).

#### 3.2.2. Bacterial Biofilm Assays

**Figure 4:**
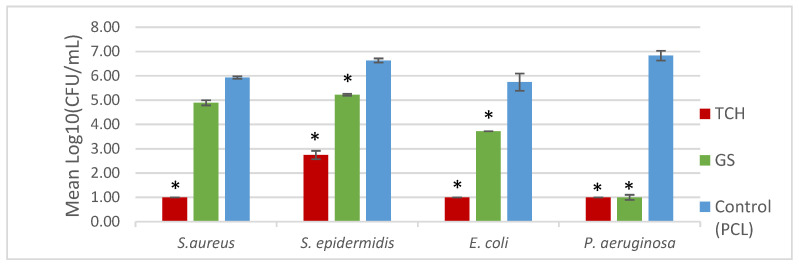
Biofilm formation on the surface of the mesh. * indicates the results that were statistically relevant.

**Figure 5:**
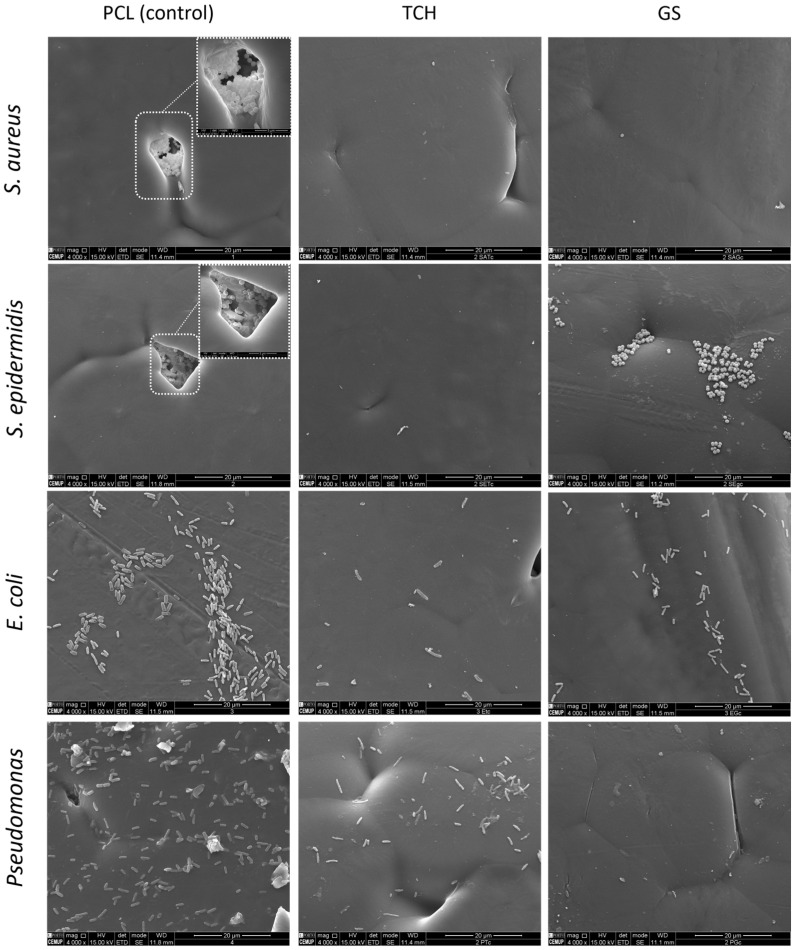
Scanning Electron Microscopy (SEM) of medical-grade PCL meshes and PCL-TCH and PCL-GS meshes.

Regarding the bacterial biofilm assays, Figure 4 represents the values for the CFU count, and Figure 5 shows the SEM images obtained. A lower CFU/mL value, and as a consequence, a lower log10 CFU/mL value, corresponds to reduced biofilm formation. A *t*-test was performed on the obtained values to determine the statistical significance of each result via the evaluation of the *p*-value. The SEM images illustrate the antibiofilm activity of the antibiotics, in accordance with the results of the log_10_ (CFU)/mL count.

## 4. Discussion

With this research, it was possible to create drug-coated fibers of ultra-fine diameters through a low-cost manufacturing technique that could be used to produce medical devices, preventing the possible infections that can occur with their implantation. Contrary to other AM techniques, MEW enables the printing of ultrafine fibers with controlled deposition and geometry without the use of toxic solvents since it is used as a polymer melt instead of the solution. That way, it is possible to print medical devices with very small dimensions, for example, meshes for treating pelvic organ prolapse [26], and depending on how small the fibers printed are, they can be used as coatings for metallic implants [27]. The application of MEW to this field has not been thoroughly investigated, having a wide range of potential applications.

It was possible to incorporate TCH and GS in medical-grade PCL meshes through submersion in 1% (*w*/*w*) antibiotic solutions, as shown in the infrared spectra obtained from FTIR analysis. This technique uses infrared light to allow for an analytical evaluation of organic, polymeric, and inorganic materials. Being a form of vibrational spectroscopy, the molecule-specific spectral bands in vibrational spectra allow for the characterization of the biochemical composition, which is achieved via the analysis of the FTIR peaks that are specific to chemical bonds or single functional groups in the molecule [28,29,30,31]. The results obtained show that PCL-TCH meshes had a more inhibitory effect for all bacteria except *P. aeruginosa*, where the growth inhibition was greater with PCL-GS meshes. Tetracyclines have a broad-spectrum antibiotic action with properties considered ideal for antibiotic drugs, having activity against Gram-positive pathogens, including *S. aureus* [32]. *S. aureus* ATCC 43300, as well as *S. epidermidis* ATCC984, are susceptible to tetracycline [33,34]. Both the inhibition zone diameters obtained for these two strains and the decrease in biofilm formation (Figure 4), which can be seen in the SEM images (Figure 5), confirm the susceptibility to TCH. This *S. aureus* strain is resistant to gentamicin [35], and at the concentration of 1% *w*/*w*, this resistance was confirmed using the zone inhibition assay. It is impossible to conclude this from the bacterial biofilm assay, given that the results were not statistically relevant. However, the PCL-GS mesh created a small inhibition zone against the *S. epidermidis* strain (Figure 3 and Table 2) in addition to a reduction in the biofilm (Figure 4). It is to be noted that in the SEM images of the control meshes, both *S. aureus* and *S. epidermidis* were not visible on all the surfaces because the bacteria were lodged in gaps on the mesh surface; the reason why is that it seems as if the PCL-GS meshes have a higher presence of these bacteria. Earlier studies have already demonstrated that *E. coli* ATCC 25922 is susceptible to gentamicin [36], and the results indicate that it maintains susceptibility, even at a concentration of 1% *w*/*w* as can be seen in the SEM images (Figure 5). The inhibition zone created by the PCL-TCH mesh and its low log10 (CFU/mL) count indicates susceptibility to this antibiotic at 1% *w*/*w* concentration, as shown before for different concentrations [36]. Finally, regarding *Pseudomonas aeruginosa* ATCC 27853, the values of the diameter of the inhibition zone obtained and the reduced biofilm formation suggest that this strain is susceptible to the PCL-GS mesh at the tested concentration of GS, in accordance with results obtained in previous studies [37,38]. In the case of the PCL-TCH mesh, even though there is a small inhibition zone diameter, the bacterial biofilm assays suggest that this strain might present low susceptibility to the antibiotic at this concentration.

## 5. Conclusions

This work demonstrated the potential of combining a MEW 3D printing technique that allows for the controlled deposition of fibers with small diameters with the antibacterial action of different drugs, in this case, TCH and GS.

TCH and GS were successfully incorporated into medical-grade PCL meshes via submersion in antibiotic solutions, as confirmed by FTIR analysis. The in vitro microbiological assays show that these combinations of PCL-TCH and PCL-GS maintained the antibacterial effect of the antibiotics, inhibiting the biofilm formation of the tested strains, especially the *P. aeruginosa* strain. This is illustrated in the SEM images, showing a decrease in the bacteria population on the surface of the PCL-TCH and PCL-GS meshes.

The printing of fibers with MEW and their subsequent incorporation with antibiotics in an immersion coating can be applied to the development of medical devices, such as catheters, and potentially used to coat other types of devices, such as prosthetics, given the small diameters that can be obtained. Therefore, it can be applied to the prevention and treatment of DRIs. Further research is needed to confirm/extend the results obtained, particularly, more extensive tests relating to the materials incorporated with the antimicrobials, to confirm their presence and concentration, and the testing of different antimicrobial concentrations and different fiber diameters.

## Figures and Tables

**Table 1:** MEW meshes’ printing parameters.

Temperature (°C)	Voltage (kV)	Nozzle’s Height (mm)	Collector’s Speed (mm/min)
200	7.0	3.0	700

**Table 2:** Mean diameters of the inhibition zones of control (PCL) PCL-TCH and PCL-GS meshes.

	*S. aureus*	*S. epidermidis*	*E. coli*	*P. aeruginosa*
Control	0 mm	0 mm	0 mm	0 mm
PCL-TCH	20 ± 2 mm	24 ± 4 mm	12 ± 3 mm	5 ± 1 mm
PCL-GS	0 mm	7 ± 2 mm	8 ± 2 mm	15 ± 3 mm

## Data Availability

The original contributions presented in the study are included in the article. Further inquiries can be directed to the corresponding author.
